# Machine learning on normalized protein sequences

**DOI:** 10.1186/1756-0500-4-94

**Published:** 2011-03-31

**Authors:** Dominik Heider, Jens Verheyen, Daniel Hoffmann

**Affiliations:** 1 Department of Bioinformatics, Center of Medical Biotechnology, University of Duisburg-Essen, Universitaetsstr. 2, 45117 Essen, Germany; 2 Institute of Virology, University of Cologne, Fuerst-Pueckler-Str. 56, 50935 Cologne, Germany

## Abstract

**Background:**

Machine learning techniques have been widely applied to biological sequences, e.g. to predict drug resistance in HIV-1 from sequences of drug target proteins and protein functional classes. As deletions and insertions are frequent in biological sequences, a major limitation of current methods is the inability to handle varying sequence lengths.

**Findings:**

We propose to normalize sequences to uniform length. To this end, we tested one linear and four different non-linear interpolation methods for the normalization of sequence lengths of 19 classification datasets. Classification tasks included prediction of HIV-1 drug resistance from drug target sequences and sequence-based prediction of protein function. We applied random forests to the classification of sequences into "positive" and "negative" samples. Statistical tests showed that the linear interpolation outperforms the non-linear interpolation methods in most of the analyzed datasets, while in a few cases non-linear methods had a small but significant advantage. Compared to other published methods, our prediction scheme leads to an improvement in prediction accuracy by up to 14%.

**Conclusions:**

We found that machine learning on sequences normalized by simple linear interpolation gave better or at least competitive results compared to state-of-the-art procedures, and thus, is a promising alternative to existing methods, especially for protein sequences of variable length.

## Background

Statistical methods and machine learning techniques, such as linear regression (LR) [1], decision trees (DTs) [2], artificial neural networks (ANNs) [3], support vector machines (SVMs) [4], and random forests (RFs) [5] have been widely applied in biomedical pattern classification, for instance in the prediction of HIV drug resistance and protein function. In several studies, the amino acid or DNA sequences were encoded by descriptors, which substitute each nucleotide or amino acid with a numerical value [6]. Some examples of descriptors are hydrophobicity, molecular weight or isoelectric point. Other studies represent a sequence by its mutations compared to the wild type sequence [7]. Yet another possible representation is the use of the standard orthonormal representation [8] or sparse encoding [9], a vector containing twenty indicator variables (one for each amino acid) for each sequence position, resulting in a matrix containing the amino acid distributions for each position within the input sequence [10].

A drawback of conventional machine learning algorithms is that they need a fixed input length, and, consequently, cannot be easily applied to data which varies in its dimension/length, as is often the case for protein sequences. One possible remedy are SVMs with string kernels [11,12]. Kernel functions return the inner product between the mapped data points in a higher dimensional space, and the special class of string kernels tries to match alignments of subsequences to build a higher dimensional feature space in which the sequences can be separated [13]. Another possible solution is the application of multiple sequence alignments [14] or multiple pairwise alignments to a reference sequence [15]. In these approaches, missing values are either filled by a specifically defined value, or by the most common value. However, this introduces some artificial information that can bias predictions.

We developed another effective, though conceptionally more simple solution, that is to linearly normalize the data to a fixed length as a preprocessing step and to subsequently apply machine learning methods to classify the data [16]. This interpolation procedure has already been successfully applied to coreceptor usage prediction in HIV-1 [17] and functional protein classification [18]. A particularly relevant application is in the prediction of HIV-1 drug resistance. Anti-retroviral treatment regimens can sufficiently suppress viral replication in HIV infected patients and prevent the progression of the disease. One of the factors contributing to the progression of the disease despite ongoing antiretroviral treatment is the emergence of drug resistance: The high mutation rate of HIV can lead to a fast adaptation of the virus under drug pressure, thus to the evolution of drug-resistant variants and failure of antiretroviral treatment. Some of the resistant strains show insertions in the drug targets HIV-1 protease and reverse transcriptase [19,20].

The focus of the current study is to compare the simple linear interpolation [16] with non-linear normalization procedures in order to evaluate the performance in subsequent classification. To this end, we tested seven HIV protease inhibitors (PIs), six HIV nucleoside reverse transcriptase inhibitors (NRTIs), three HIV nonnucleoside reverse transcriptase inhibitors (NNRTIs) and one HIV maturation inhibitor (MI) datasets. PIs prevent viral replication by inhibiting the activity of HIV-1 protease, an enzyme used by the viruses to cleave nascent polypeptides into functional proteins. They are designed to have a high affinity to the catalytic center of the HIV protease, thereby hampering its enzymatic activity. NRTIs and NNRTIs inhibit the activity of the reverse transcriptase (RT). NRTIs are nucleoside analogs, and thus, compete for the RT with the natural nucleosides. An incorporation of a NRTI leads to a premature termination of the viral genome replication. In contrast, NNRTIs are non-competitive inhibitors of the RT. They inhibit the movement of protein domains of the RT that is needed to carry out the process of DNA synthesis. MIs, such as Bevirimat, inhibit maturation of virus particles by preventing cleavage of precursor polyprotein gag by the HIV-1 protease.

Besides the comparison between the linear and non-linear methods, we also compare our classification results with results obtained from state-of-the-art methods. We focus on the comparison between our method and other methods that employed exactly the same publicly available datasets, namely Rhee *et al*. [7], Hou *et al*. [21], Kierczak *et al*. [22] and Heider *et al*. [23].

Rhee *et al*. used five different statistical and machine learning methods (DTs, ANNs, SVMs, least-squares regression and least angle regression) to predict drug resistance in HIV-1 [7] for 16 drugs. A sequence was represented by its set of mutations compared to the wild type sequence. Hou *et al*. developed a machine learning approach for the prediction of PI resistance based on SVMs [21], but in contrast to Rhee *et al*., they used structure-derived descriptors. Kierczak *et al*. [22] developed a set-based model considering physico-chemical changes of mutated sequences compared to the wildtype strain to predict NRTI and NNRTI resistance. Heider *et al*. [23] used a multiple sequence alignment of the p2 sequences as an input for a RF to predict Bevirimat resistance. Other published methods, e.g. [24-26] employed other datasets, and hence, their results cannot be easily compared with our method.

To check whether normalization is also advantageous in other applications, we tested two datasets dealing with protein functional class prediction [16,18], namely the classification of small GTPases and the classification of the major intrinsic protein family (MIP). Small GTPases are small monomeric proteins that can act as "molecular switches" due to their ability to bind and hydrolyze GTP. In its GTP-bound form a small GTPase is active, whereas a hydrolysis of GTP to GDP converts the protein into its inactive conformation [27]. Small GTPases are involved in many cellular processes including differentiation, cell division, vesicular transport, nuclear assembly, and control of the cytoskeleton. The involvement of a variety of Ras superfamily proteins in human tumorigenesis makes these proteins interesting subjects in cancer research, and hence, the identification and functional characterization of novel GTPases is an important topic in molecular cell biology [28]. The MIPs are a large family of different types of membrane channels, e.g. aquaporines [29].

## Results and discussion

**Figure 1:**
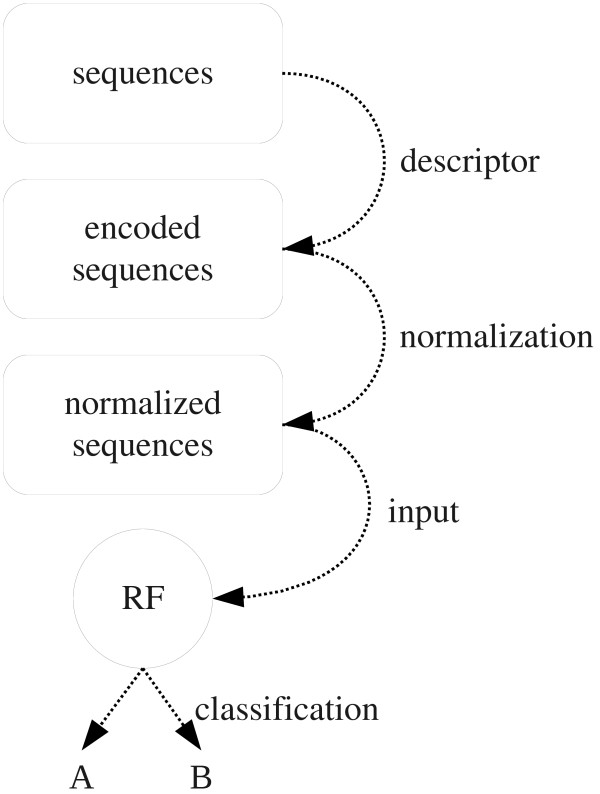
**Workflow of the applied procedure**. The protein sequences are first encoded as vectors of numerical descriptor values, e.g. with the hydropathy values of Kyte and Doolittle [30]. These vectors are normalized to a fixed length by applying the described interpolation methods. Finally, the normalized encoded sequences are used as input for the random forests in the classification.

The workflow applied in the current study is shown in Figure 1. The sequences are encoded with the hydropathy descriptor [30] and subsequently normalized to a fixed length with the mentioned interpolation methods. The normalized sequences are used to train the RF model. We also tested the *net charge*, molecular weight and isoelectric point as descriptors to encode the sequences. The relative performance of each normalization procedure in comparison to each other is quite similar for all descriptors. However, the hydropathy descriptor works best with regard to the prediction performance, and thus we only show the results of the hydropathy descriptor in the following.

HIV-1 protease and reverse transcriptase have rather well conserved lengths of 99 and 240 amino acids, respectively [7]. However, some of the protease and reverse transcriptase sequences have insertions/deletions. In constrast, sequence lengths of the GTPases and the MIP datasets are highly variable (see Table 1) [16,23].

**Table 1:** Summary of the datasets

dataset	# sequences	positive samples	negative samples	length
APV	768	61%	39%	99.70 ± 1.24%
ATV	329	48%	52%	99.59 ± 1.06%
IDV	827	51%	49%	99.68 ± 1.23%
LPV	517	45%	55%	99.73 ± 1.22%
NFV	844	40%	60%	99.67 ± 1.22%
RTV	795	49%	51%	99.71 ± 1.24%
SQV	826	60%	40%	99.69 ± 1.23%

3TC	633	31%	69%	240.87 ± 2.33%
ABC	628	29%	71%	240.54 ± 4.20%
AZT	630	52%	48%	240.87 ± 2.33%
d4T	630	54%	46%	240.54 ± 4.20%
ddI	632	49%	51%	240.87 ± 2.33%
TDF	353	67%	33%	240.72 ± 1.88%

DLV	732	64%	36%	241.28 ± 1.49%
EFV	734	62%	38%	241.32 ± 1.49%
NVP	746	57%	43%	241.30 ± 1.48%

BVM	155	28%	72%	20.77 ± 2.07%

GTP	1435	46%	54%	232.18 ± 22.37%

MIP	49	39%	61%	261.41 ± 21.47%

The table summarizes number of sequences within each dataset, percentages of positive and negative samples, average lengths ± standard deviations in percent.

Table 2 shows AUC values of the predictions, Table 3 the results of the Wilcoxon Signed-Rank tests (significance level *α *= 0.05). In the case of a maximal (*max*) interpolation factor, the linear interpolation method outperforms the other interpolation methods in most of the datasets, except for 3TC, NVP and BVM. Using the most frequent sequence length as interpolation factor (*most*), the picture is less clear. The linear interpolation works best for the datasets ATV, AZT, ddI, TDF, DLV, EFV, GTP and MIP and the fmm interpolation for the datasets NFV, RTV and SQV. In most of the datasets *max *and *most *interpolation do not perform significantly different according to the Wilcoxon Signed-Rank tests.

**Table 2:** Prediction results

Drug	linear max most	splines max most	fmm max most	periodic max most	natural max most
APV	0.934 ± 0.001	0.929 ± 0.002	0.928 ± 0.001	0.927 ± 0.001	0.928 ± 0.001
	0.932 ± 0.001	0.934 ± 0.001	0.932 ± 0.002	0.933 ± 0.001	0.933 ± 0.001
ATV	0.936 ± 0.002	0.917 ± 0.003	0.920 ± 0.002	0.919 ± 0.002	0.920 ± 0.002
	0.928 ± 0.002	0.915 ± 0.003	0.919 ± 0.003	0.918 ± 0.003	0.920 ± 0.003
IDV	0.972 ± 0.001	0.968 ± 0.001	0.968 ± 0.001	0.968 ± 0.001	0.968 ± 0.001
	0.970 ± 0.001	0.970 ± 0.001	0.971 ± 0.001	0.971 ± 0.001	0.972 ± 0.001
LPV	0.964 ± 0.001	0.963 ± 0.001	0.963 ± 0.001	0.962 ± 0.001	0.963 ± 0.001
	0.963 ± 0.001	0.964 ± 0.001	0.963 ± 0.001	0.963 ± 0.001	0.964 ± 0.001
NFV	0.941 ± 0.001	0.938 ± 0.001	0.940 ± 0.001	0.940 ± 0.001	0.940 ± 0.001
	0.939 ± 0.001	0.943 ± 0.001	0.947 ± 0.001	0.946 ± 0.001	0.945 ± 0.001
RTV	0.984 ± 0.001	0.980 ± 0.001	0.981 ± 0.001	0.981 ± 0.001	0.981 ± 0.001
	0.983 ± 0.001	0.986 ± 0.001	0.986 ± 0.001	0.986 ± 0.001	0.986 ± 0.001
SQV	0.955 ± 0.001	0.950 ± 0.001	0.951 ± 0.001	0.951 ± 0.001	0.951 ± 0.001
	0.952 ± 0.001	0.953 ± 0.001	0.957 ± 0.001	0.955 ± 0.001	0.956 ± 0.001

3TC	0.933 ± 0.002	0.936 ± 0.002	0.939 ± 0.002	0.938 ± 0.002	0.939 ± 0.002
	0.927 ± 0.003	0.934 ± 0.002	0.937 ± 0.002	0.937 ± 0.002	0.937 ± 0.003
ABC	0.916 ± 0.002	0.906 ± 0.002	0.909 ± 0.003	0.909 ± 0.002	0.909 ± 0.002
	0.914 ± 0.003	0.910 ± 0.003	0.918 ± 0.003	0.919 ± 0.002	0.918 ± 0.003
AZT	0.908 ± 0.002	0.890 ± 0.002	0.894 ± 0.002	0.893 ± 0.002	0.894 ± 0.002
	0.908 ± 0.002	0.898 ± 0.002	0.905 ± 0.002	0.903 ± 0.002	0.904 ± 0.002
d4T	0.903 ± 0.002	0.886 ± 0.002	0.889 ± 0.002	0.889 ± 0.002	0.889 ± 0.002
	0.900 ± 0.002	0.892 ± 0.002	0.901 ± 0.002	0.899 ± 0.002	0.901 ± 0.002
ddI	0.853 ± 0.003	0.829 ± 0.003	0.837 ± 0.003	0.836 ± 0.003	0.836 ± 0.002
	0.852 ± 0.003	0.841 ± 0.003	0.846 ± 0.003	0.839 ± 0.003	0.844 ± 0.003
TDF	0.832 ± 0.004	0.808 ± 0.005	0.817 ± 0.004	0.818 ± 0.005	0.816 ± 0.005
	0.825 ± 0.005	0.812 ± 0.005	0.813 ± 0.005	0.814 ± 0.005	0.813 ± 0.005

DLV	0.901 ± 0.002	0.888 ± 0.002	0.891 ± 0.002	0.891 ± 0.002	0.891 ± 0.002
	0.898 ± 0.002	0.881 ± 0.002	0.882 ± 0.002	0.883 ± 0.002	0.883 ± 0.002
EFV	0.932 ± 0.002	0.921 ± 0.002	0.928 ± 0.002	0.929 ± 0.002	0.928 ± 0.002
	0.925 ± 0.002	0.911 ± 0.002	0.915 ± 0.002	0.919 ± 0.002	0.915 ± 0.002
NVP	0.917 ± 0.002	0.910 ± 0.002	0.916 ± 0.002	0.917 ± 0.002	0.916 ± 0.002
	0.908 ± 0.003	0.902 ± 0.003	0.906 ± 0.003	0.909 ± 0.003	0.906 ± 0.003

BVM	0.918 ± 0.002	0.932 ± 0.002	0.932 ± 0.002	0.923 ± 0.003	0.933 ± 0.002

GTP	0.981 ± 0.001	0.979 ± 0.001	0.978 ± 0.001	0.977 ± 0.001	0.979 ± 0.001
	0.980 ± 0.001	0.979 ± 0.001	0.979 ± 0.001	0.976 ± 0.001	0.979 ± 0.001

MIP	0.815 ± 0.010	0.789 ± 0.013	0.789 ± 0.011	0.787 ± 0.016	0.788 ± 0.017
	0.827 ± 0.012	0.815 ± 0.014	0.813 ± 0.014	0.816 ± 0.013	0.812 ± 0.013

AUC ± standard deviations with *max *representing the maximal occuring sequence length within a dataset, *most *the most frequent sequence length in a dataset. For BVM *most *and *max *are the same.

**Table 3:** Wilcoxon Signed-Rank tests

method	APV	ATV	IDV	LPV	NFV	RTV	SQV	3TC	ABC	AZT	D4T	DDI	TDF	DLV	EFV	NVP	BVM	GTP	MIP
linear	*	*	*	*	*	*	*		*	*	*	*	*	*	*	*	*	*	*
splines																			
fmm								*									*		
periodic								*								*			
natural								*									*		

linear	*	*		*						*		*	*	*	*		*	*	*
splines																			
fmm					*	*	*	*	*		*						*		
periodic								*	*							*			
natural			*	*				*	*		*						*		

Wilcoxon Signed-Rank tests on the AUC distributions. The method performing best and having significantly higher AUC values (*α *= 0.05) is marked with *. When a test is not significant more than one method is marked. The upper part shows the result of the max-interpolation, the lower part the results of the most-interpolation.

Rhee *et al*. used five different statistical and machine learning methods (DTs, ANNs, SVMs, least-squares regression and least angle regression) to predict drug resistance in HIV-1 [7]. In contrast to our encoding procedure, a sequence was represented by its set of mutations compared to the wild type sequence. Hou *et al*. developed a machine learning approach for the prediction of PI resistance (APV, ATV, IDV, LPV, NFV, RTV and SQV) based on SVMs [21], but in contrast to Rhee *et al*., they used structure-derived descriptors. Their method had a higher accuracy compared to the results of Rhee *et al*. Table 4 shows a comparison of the prediction accuracy for the best models of Rhee *et al*., Hou *et al*. and the best model based on the methods used in the current study. Judged from the comparison of the results, normalization of sequences seems in general to improve classification performance, except for APV (89% vs. 88% accuracy) and SQV (89% vs. 89% accuracy). However, we used the complete datasets whereas the results of Rhee *et al*. and Hou *et al*. are based on the best models for specific subsets, e.g. for APV they report the results of the TSM subset (the best model of Rhee *et al*. for the complete APV dataset reached only an accuracy of 82%). For the NRTI and NNRTI datasets, our procedure yielded higher accuracy compared to the results from Rhee *et al*. for all datasets, except for 3TC (90% vs. 90% accuracy) and for NVP (91% vs. 87% accuracy). As neither Rhee *et al*. nor Hou *et al*. provided standard deviations for the prediction accuracy, we cannot perform a statistical comparison to warrant that our results are significantly better for all datasets. To assess the relative impact of normalization and actual machine learning method, we also applied artificial neural networks in accordance to Rhee *et al*. The results are worse compared to the results obtained with the RF classification, but still better than the results of Rhee *et al*., thus justifying sequence normalization as a preprocessing step.

**Table 4:** Comparison of the prediction accuracy

drug	Rhee *et al.*	Hou *et al.*	this study
APV	84%	**89%**	88%
ATV	77%	86%	**88%**
IDV	79%	86%	**93%**
LPV	81%	91%	**92%**
NFV	82%	87%	**91%**
RTV	89%	93%	**95%**
SQV	84%	**89%**	**89%**

3TC	**90%**	*	**90%**
ABC	77%	*	**88%**
AZT	76%	*	**84%**
d4T	78%	*	**84%**
ddI	75%	*	**79%**
TDF	73%	*	**79%**

DLV	84%	*	**87%**
EFV	87%	*	**88%**
NVP	**91%**	*	87%

*: Hou *et al*. used only the PI datasets [21].

Kierczak *et al*. [22] provide AUC values with standard deviations, so their results are directly comparable to our results (see Table 5). Our results led to substantially better results for the drugs ABC, d4T, DLV, NVP, slighty better results for the drugs AZT and ddI and slightly worse results for the drugs 3TC and TDF. Kierczak *et al*. do not provide results for the drug EFV. Our results show smaller standard deviations compared to the results of Kierczak *et al*., which are based on a rough set-based model [22]. For BVM resistance classification, we obtained an AUC of 0.933 ± 0.002 with machine learning on normalized sequences, which is slightly, but significantly higher (according to Wilcoxon Signed-Rank test at *α *= 0.05) than that (0.927 ± 0.001) obtained with aligned sequences of the HIV-1 p2 sequences as input [23], thus again justifying sequence normalization as a preprocessing step. The best AUCs for the protein functional class prediction of the small GTPases and the MIPs are 0.981 ± 0.001 and 0.827 ± 0.012, respectively, which are in accordance with our recently published results [16,18].

**Table 5:** AUC comparison

drug	Kierczak *et al.*	this study
3TC	0.95 ± 0.03	0.94 ± 0.00
ABC	0.83 ± 0.05	0.92 ± 0.00
AZT	0.89 ± 0.05	0.91 ± 0.00
d4T	0.85 ± 0.06	0.90 ± 0.00
ddI	0.82 ± 0.08	0.85 ± 0.00
TDF	0.85 ± 0.05	0.83 ± 0.00

DLV	0.76 ± 0.06	0.90 ± 0.00
EFV	*	0.93 ± 0.00
NVP	0.85 ± 0.05	0.92 ± 0.00

AUC ± standard deviations.

*: Kierczak *et al*. analyzed the NRTI and NNRTI datasets except EFV [22].

**Figure 2:**
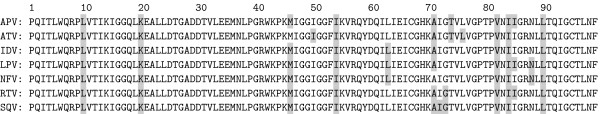
**Most important sequence positions for the PI classification**. Sequences of HIV-1 protease with the ten most important positions marked in gray.

As mentioned before, RFs are able to identify the most important positions for the classification process [31]. We studied importance of sequence positions for each of our datasets. Five positions (L10, K20, I54, V82, L90) in the HIV-1 protease are found in the top ten list of most important mutations for the correct classification of each protease inhibitor and two further known mutations (M46, A71) are found in almost all datasets (see Figure 2). Mutations at three positions - L10, K20 and A71 - are known as compensatory, i.e. compensating for the loss of enzyme activity due to major protease mutations. These findings might be explained by the origin of data from patients having experienced multitherapy failures and thus developed highly adapted viral strains. This would be in line with the findings that protease inhibitor specific protease mutations belong to the twenty most important positions for the classification process (APV: 32, 76; SQV: 48 and LPV: 76).

The ten most important resistance mutations of each protease inhibitor (involving protease positions (50, 63, 73, 74, 76, 85 and 88)) are also in accordance with previous *in vitro *and *in vivo *findings (APV: 74 [32], LPV: 63 [33], ATV: 50 [34], NFV: 88 [35]).

The scoring of protease position 76 in terms of predicted ATV resistance is interesting, since it is well known that protease mutation 76V, which confers resistance to LPV and APV, re-sensitizes these HIV isolates to the protease inhibitors SQV and ATV. Indeed, HIV isolates carrying protease mutation 76 V accumulate in the group of susceptible to ATV therapy regimens, which therefore explains the prediction of susceptible rather than resistant. This effect of re-sensitization has been considered mainly in rule-based HIV drug resistance interpretation tools, but has failed so far to reach statistical significance in machine learning approaches. For the NRTIs and NNRTIs the RFs also identified major known resistant mutations such as M41, L74, M184, L210 and K219 [36].

The normalization of the sequence length for the prediction of drug resistance allows to analyze HIV protease sequences carrying insertions/deletions. Insertions in the protease are sometimes observed in HIV isolates failing PI therapies. It has been shown in a recent study [20] that the prevalence of insertions has increased significantly in the last years. Furthermore, it has been shown in an earlier study [19] that RT insertions are frequently found in heavily-treated patients, which can enhance NRTI resistance and may improve viral fitness. Our classification procedure is able to classify these recently published protease and reverse transcriptase insertions in terms of resistance correctly.

## Conclusions

In most of the cases studied here, linear interpolation gave superior AUC values in comparison with non-linear schemes. In some cases, other interpolation methods lead to slightly, but significantly higher AUCs, e.g. for NFV, RTV, SQV, 3TC, ABC and BVM. Interpolating sequence lengths in combination with hydropathy as a descriptor and RFs led to at least competitive results compared to other methods [7,21-23]. Although sequence length variations are rare in the case of HIV-1 protease and reverse transcriptase, there are some insertions and deletions known, and these can be handled consistently with the proposed procedure. Our method is able to correctly predict drug resistance in HIV-1 isolates carrying insertions in the protease [20]. Moreover, the GTPases and the MIP datasets show a high sequence variability, which can be easily handled with our proposed scheme. As demonstrated, there is no significant performance difference between the *most *and the *max *interpolation. Finally, the proposed normalization procedure based on a simple linear interpolation is not limited to studies dealing with HIV-1 drug resistance or classification of small GTPases. Other applications may address e.g. protein-protein interaction prediction [37,38], prediction of protein cellular attributes [39], protein localization prediction [40] and protein remote homology detection [41]. The current study provides evidence for the reliability of the simple linear interpolation for handling varying protein sequence lengths in a broader range of biomedical classification studies.

## Methods

### Data

The data was gathered from two classification studies of HIV-1 drug resistance and two protein functional class prediction studies [7,16,23]. We analyzed the drug target protein sequences for resistance (= negative) or susceptibility (= positive) to the corresponding drugs. These drugs include seven protease inhibitors (PIs) Amprenavir (APV), Atazanavir (ATV), Indinavir (IDV), Lopinavir (LPV), Nelfinavir (NFV), Ritonavir (RTV), Saquinavir (SQV), the six nucleoside reverse transcriptase inhibitors (NRTIs) Lamivudine (3TC), Abacavir (ABC), Zidovudine (AZT), Stavudine (d4T), Didanosine (ddI), Tenofovir (TDF), the three nonnucleoside reverse transcriptase inhibitors (NNRTIs) Delavirdine (DLV), Efavirenz (EFV), Nevirapine (NVP) and the maturation inhibitor Bevirimat (BVM). Furthermore, we analyzed protein sequences for membership or non-membership in the functional class of small GTPases and MIP, respectively. These datasets contained protein sequences belonging to the specific family (= positive) and proteins that do not belong to the family (= negative) [16]. Table 1 shows a summary of the datasets used in the current study. The ratio of the positive to the negative class is at least 1:3 (except for ABC, 3TC and BVM). The cut-offs of the *IC*_50 _values between susceptible and resistant sequences are in accordance with Rhee *et al*. [7] and Heider *et al*. [23].

### Descriptor set

It has been shown to be helpful to associate with each amino acid a numerical "descriptor" value, for instance a value that captures a physico-chemical property of this amino acid, instead of treating sequences of amino acids as strings of characters. The selection of the descriptor set is the most critical part in classification [6,42], and, in general, physico-chemical descriptors outperform simpler descriptors [43]. In particular the hydropathy index of Kyte and Doolittle [30] has proven in several studies to be a powerful descriptor [44-48]. Therefore, we used this index to encode the amino acids in the protein sequences. Moreover, we also tested *net charge*, molecular weight and isoelectric point as descriptors for encoding of the amino acids.

### Normalization procedures

The HIV-1 protease (PR) sequences, the HIV-1 reverse transcriptase (RT) sequences, the HIV-1 p2 sequences and the protein sequences for functional classification were normalized to the maximally (*max*) occurring sequence lengths as well as to the most frequent sequence lengths (*most*). We used five different normalization procedures, the simple linear interpolation and four spline interpolations implemented in different R-packages (http://www.r-project.org/):

• simple linear interpolation (linear)

• cubic spline interpolation (splines)

• spline interpolation of Forsythe [49] (fmm)

• periodic spline interpolation (periodic)

• natural spline interpolation (natural)

The linear interpolation connects two known data points, (*x*_0_, *y*_0_) and (*x*_1_, *y*_1_), with a straight line. *x_i _*indicates the sequence position and *y_i _*indicates the corresponding value of the amino acid at position *x_i_*. The y value of a value × in the interval [*x*_0_, *x*_1_] is given by(1)

Linear interpolation on a set of data points (*x*_0_, *y*_0_), (*x*_1_, *y*_1_),..., (*x_n_*, *y_n_*) is defined as the concatenation of linear interpolants between each pair of successive data points. The cubic spline interpolation uses piecewise cubic polynomials between the data points. The spline interpolation of Forsythe [49] is a variant of cubic spline interpolation with the cubic passing exactly through the four points at each end of a sample (here: the four encoded amino acids at each end of a protein). The periodic spline interpolation fits a curve that fulfills periodic boundary conditions, i.e. the spline curve has the same first and second derivative at its endpoints. For the natural spline interpolation, the natural boundary conditions are fulfilled. All interpolation methods result in continuous curves connecting all known data points. However, the progressions of the curves differ from each other.

**Figure 3:**
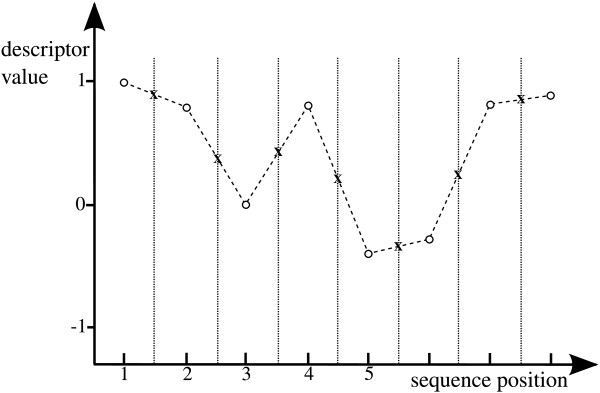
**Simple linear interpolation**. Circles mark descriptor values for the amino acids, Xs represent interpolated values. In this example, the sequence is interpolated from 8 to 15 values.

The *normalization factor *is defined as the number of samples taken (by equal interval) from the aforementioned curves to generate an input for the subsequent classification. In Figure 3 the application of the simple linear interpolation from 8 to 15 values is demonstrated with a fictitious descriptor mapping the twenty amino acids to numerical values between -1 and 1. Starting with sequence *s *= *PLAIRNIQ *the descriptor encodes *s *into the vector . Applying the simple linear interpolation with a normalization factor *n *= 15 results in the interpolated vector . A fragment of the R-code for creating a Forsythe interpolation is shown here:

# for all samples in the data set

for(i in 1:number_of_samples) {

   y = data[i,]                                 # pick sequence i

   x = seq(1:length(y))

   f = splinefun(x,y, method="fmm")            # create interpolation function f

   stepsize = length(y)/normalization_factor   # new stepsize

   x.new = c()                                 # new resulting × values

   for(n in 1:normalization_factor){

      x.new = c(x.new, n*stepsize)

   }

   y.new = f(x.new)                           # calculation of new y values

   data.new = cbind(data.new, y.new)         # adding new sample to the new dataset

}

### Classification

We trained random forests (RF) [31] as implemented in the R package randomForest (http://www.r-project.org/) for the classification. Earlier studies have shown that RFs are excellent non-linear classifier, which are highly stable and robust in comparison to other classifiers [50]. They consist of a set of independent decision trees whose outputs are combined to generate a final decision. In our application, each RF consisted of 2000 randomly and independently grown decision trees. When using the trained RF for prediction, an unseen sequence was assigned to the class voted for by at least 50% of the trees.

RFs provide an importance analysis, which can be used to identify the most important positions for the classification process. The importance measures the decrease in prediction accuracy, when the corresponding variable is permuted [31]. As the importance measure might be affected by correlated positions [51], we calculated the pairwise correlation of each sequence position with each other position. It turned out that the importance measurement is not affected by correlation.

### Cross-validation

The RFs were validated using 100-fold leave-one-out [52] validation to evaluate the average prediction sensitivity, specificity, and accuracy (see formulas below) and the ability to generalize to unseen sequences. The sensitivity, specificity, and accuracy were calculated according to:(2)(3)(4)

with true positives *TP*, false positives *FP*, false negatives *FN *and true negatives *TN*. Furthermore, we calculated the Receiver Operating Characteristics (ROC) curve [53] and the corresponding area under the curve (AUC) with ROCR [54]. The ROC curve is built by plotting sensitivity and specificity against each other for every possible cut-off between the two classes.

### Statistical comparison

All interpolation procedures were compared by applying Wilcoxon Signed-Rank tests [55] on the AUC distributions from the 100-fold leave-one-out cross-validation runs according to Demsar [56]. The null hypothesis was that there are no differences between the compared classifiers.

## Competing interests

The authors declare no competing interests.

## Authors' contributions

DH* has developed the research concept, carried out computational analyses and drafted the manuscript.

JV and DH have interpreted results and revised the manuscript.

All authors read and approved the final manuscript.
